# Entwicklung und Bedeutung der programmierten Ventrikelstimulation bei der koronaren Herzerkrankung und dilatativen Kardiomyopathie

**DOI:** 10.1007/s00399-024-01003-3

**Published:** 2024-02-29

**Authors:** Bernd-Dieter Gonska

**Affiliations:** Karlsruhe, Deutschland

**Keywords:** Programmierte Ventrikelstimulation, Koronare Herzkrankheit, Dilatative Kardiomyopathie, Induzierbarkeit, Arrhythmiekontrolle, Programmed ventricular stimulation, Coronary artery disease, Dilative cardiomyopathy, Inducibility, Arrhythmia control

## Abstract

Die programmierte Ventrikelstimulation (PVS) wurde in den 80er-Jahren des letzten Jahrhunderts in die Klinik eingeführt. Ziel ist es, die elektrische Vulnerabilität des Ventrikelmyokards zu prüfen und unabhängig von der Spontanvariabilität ventrikulärer Arrhythmien diese unter kontrollierten Bedingungen auszulösen. Als spezifisch gilt die Induzierbarkeit einer monomorphen anhaltenden ventrikulären Tachykardie (VT). Das Stimulationsergebnis wird jedoch durch die Art der kardialen Erkrankung eingeschränkt – nichtischämische Erkrankungen haben eine geringe Induzierbarkeit und Reproduzierbarkeit − und die Therapiekontrolle einer pharmakologischen antiarrhythmischen Therapie ist unzuverlässig. Eine prognostische Aussage hinsichtlich des Risikos eines plötzlichen Herztodes ist unsicher. Der heutige Einsatz besteht in der Kontrolle nach einer VT-Katheterablation und vorher nachgewiesener VT-Induzierbarkeit.

## Abstract (en)

Programmed ventricular stimulation (PVS), a clinical tool introduced in the early 1980s, aims to prove the electrical vulnerability of the heart and, independent of spontaneous arrhythmia variability, to trigger arrhythmias under controlled conditions. A specific response is the inducibility of monomorphic sustained ventricular tachycardia. This depends on the underlying heart disease, e.g., only for coronary artery disease but not for nonischemic diseases. The value of pharmacologic arrhythmia control as serial electrical testing is uncertain. Up to now there seems to be no prognostic value of PVS concerning sudden cardiac death. PVS is used as a tool to monitor the results of ventricular tachycardia (VT)-catheter ablation in patients who were primarily inducible.

Herzerkrankungen sind in hohem Maße durch ventrikuläre Arrhythmien belastet. Insbesondere die häufigste Herzerkrankung, die koronare Herzkrankheit (KHK) ist davon betroffen. Für Kardiomyopathien und valvulären Erkrankungen besteht ebenfalls eine hohe Rate an spontan auftretenden Arrhythmien. Der prädiktive Wert der spontanen komplexen Arrhythmien in Bezug auf das Auftreten des plötzlichen Herztodes ist bis heute in der Diskussion. Die bisherigen Untersuchungen zur dilatativen Kardiomyopathie zeigen eine hohe Rate spontan auftretender komplexer Arrhythmien. Offen bleibt die prognostische Bedeutung. Bei der hypertrophen Kardiomyopathie erscheint es insofern einfacher, als der genetische Bezug und das Ausmaß der morphologischen Veränderungen einen Hinweis auf das prognostische Risiko erlauben. Die restriktive Kardiomyopathie ist hingegen noch relativ gering untersucht. Die Daten über die arrhythmogene, rechtsventrikuläre Erkrankung erlauben keine Einschätzung.

Der plötzliche arrhythmogene Herztod betrifft ca. 50 % aller kardialen Todesfälle. Bei Patienten mit koronarer Herzkrankheit lassen sich in 60–90 % der Patienten, bei der dilatativen Kardiomyopathie bei 80–100 % im spontanen EKG und Langzeit-EKG ventrikuläre Extrasystolen nachweisen. Komplexe ventrikuläre Arrhythmien in Form von Polymorphie und repetitiver Formen werden bei 40–60 % der Patienten mit koronarer Herzkrankheit und 50–80 % der Patienten mit dilatativer Kardiomyopathie gesehen. Das Ausmaß des spontanen Arrhythmieverhaltens steht bei der koronaren Herzkrankheit in einer inversen Beziehung zur linksventrikulären Funktion. Quantität und Qualität und damit Komplexität steigen mit dem Ausmaß der linksventrikulären Funktionseinschränkung. Mehrere Untersuchungen haben auf die prognostische Bedeutung ventrikulärer Arrhythmien für das Eintreten des plötzlichen Herztodes bei der koronaren Herzerkrankung hingewiesen [1–4]. Für die nichtischämischen Herzmuskelerkrankungen besteht dieser Zusammenhang nicht. Eine prognostische Bedeutung komplexer ventrikulärer Rhythmusstörungen bei Patienten mit einer dilatativen Kardiomyopathie auch als Hinweis auf ein erhöhtes Risiko für den plötzlichen Herztod scheint nach neueren Daten nicht zu bestehen. Hierauf weisen auch kürzlich publizierte Daten wie die der DANISH-Studie [5] hin.

Die Mortalitätsrate nach einem Myokardinfarkt betrug in den Studien der 80er- und 90er-Jahre des letzten Jahrhunderts im ersten Jahr noch 10–20 %, in jedem weiteren ca. 5 % [6–8]. Etwa 50 % der Fälle waren plötzliche Herztodesfälle [7, 9].

Als Ursache des plötzlichen Herztodes kommen in ca. 80 % ventrikuläre Tachyarrhythmien in Betracht [10].

Die Aufgabe der klinischen Elektrophysiologie war und ist es, Verfahren zu entwickeln, die das Risiko im Vorfeld klären und eine Identifikation des gefährdeten Patienten ermöglichen. Die Bestimmung der elektrischen Vulnerabilität mit Hilfe der ventrikulären Stimulation wurde von Hein Wellens [11] als ein invasives Verfahren eingeführt. Zwei Indikationen für die programmierte Ventrikelstimulation ergaben sich:

1. der Versuch der Risikostratifikation im Sinne einer Primärprävention,
2. die Reproduktion einer ventrikulären Tachykardie, um eine apparative oder pharmakologische Sekundärprävention zu betreiben.

Für den Einsatz der programmierten Ventrikelstimulation spricht, dass das Verfahren unabhängig von der Spontanvariabilität der Arrhythmien ist und zweitens eine zumindest bei der koronaren Herzkrankheit eine hohe Reproduzierbarkeit hat.

Nach ersten Untersuchungen von DeBoer 1939 [12] und von Wiggers 1940 [13] wurde die frühzeitige Applikation eines elektrischen Stimulus mit einer hohen Reizintensität nach einer vorangegangenen Kammerdepolarisation zur Auslösung von Kammerflimmern beschrieben. Sie bezeichneten die hierzu erforderliche Reizintensität als *Flimmerschwelle* und den zeitlichen Abstand zum vorangegangenen Kammerkomplex als *vulnerable Phase*. Die Reizschwelle zur Auslösung von Kammerflimmern ist jedoch um ein Vielfaches höher als die zur Auslösung einer einzelnen Erregung [14].

In tierexperimentellen Studien untersuchte die Arbeitsgruppe um Bernhard Lown die Wirkung elektrischer Einzelimpulse niederer und mittlerer Intensität auf das Myokard [15].

Bei einem elektrisch stabilen Ventrikel erzeugte ein vorzeitiger Einzelstimulus nur einzelne ventrikuläre Erregungen. Bei einer Vorschädigung des Ventrikels hingegen führten vorzeitige Impulse zu kurzen Salven ventrikulärer Zusatzerregungen. Als möglicher Gradmesser für die Kammervulnerabilität fand diese Methode in der zweiten Hälfte der 70er Jahre des vergangenen Jahrhunderts Eingang in die klinische Elektrophysiologie.

Eine Vielzahl von Stimulationsprotokollen wurde bisher geschrieben, ohne dass eine Standardisierung bis heute erreicht werden konnte. Eine Übereinstimmung in allen klinisch elektrophysiologisch tätigen Zentren lag darin, dass ein Stimulationsprotokoll, bei dem die Stimulation mit doppeltem diastolischem Schwellenwert bei Sinusrhythmus und drei Frequenzen eines kammerstimulierten Grundrhythmus stattfand. Nach 8 spontanen oder jeweils 8 stimulierten QRS-Komplexen wurden 1 bis 3 Extrastimuli appliziert. Die Ankoppelung der Extrastimuli erfolgte bis zur effektiven Refraktärzeit (RFP), die fixe Ankoppelung danach mit einer Zeit von 30 ms oberhalb der RFP.

## Die programmierte ventrikuläre Stimulation zur Risikostratifikation des plötzlichen Herztodes

Die prognostische Aussagekraft des Stimulationsergebnisses ist nicht geklärt. Als spezifisches Ergebnis kann die Induzierbarkeit einer monomorphen ventrikulären Tachykardie gelten. Die Induzierbarkeit eines solchen Ergebnisses setzt pathologisch anatomisch das Vorliegen einer myokardialen Narbe voraus. Insofern kann dieses Ergebnis nur für ein kleines, gut charakterisiertes Patientenkollektiv gelten. Die reproduzierbare Induzierbarkeit nichtanhaltender ventrikulärer Tachykardien oder von Kammerflimmern stellt ein unspezifisches Ergebnis dar.

Die Auslösung einer repetitiven Kammerantwort muss daher nicht unbedingt eine Prädisposition für den plötzlichen Herztod bedeuten. Inwieweit das Stimulationsergebnis im Abstand zu einem akuten Myokardinfarkt eine prognostische Aussage erlaubt, wurde untersucht. Ein klinisch relevantes Ergebnis hat sich hieraus nicht ergeben [16–18].

Die Bedeutung der programmierten Ventrikelstimulation wurde in den 90er-Jahren des vergangenen Jahrhunderts immer wieder infrage gestellt. Beispielhaft sind folgende Arbeiten:

Marchlinski [19] untersuchte 46 Patienten innerhalb von 8 bis 60 Tagen nach einem Herzinfarkt. Bei 5 Patienten konnte eine nichtanhaltende und bei 5 Patienten eine anhaltende Kammertachykardie induziert werden. Innerhalb der Nachbeobachtungszeit von 18 Monaten verstarben 10 Patienten, 6 davon plötzlich. Lediglich ein Patient hatte eine induzierbare ventrikuläre Tachykardie. Das Stimulationsergebnis hatte demnach keine prognostische Aussage. Einzige Gemeinsamkeit der Verstorbenen war die reduzierte LVEF < 40 %. Ein vergleichbares Ergebnis wurde von Buxton [20] beschrieben, der bei 83 Patienten mit asymptomatischen nichtanhaltenden ventrikulären Tachykardien – 33 Patienten mit chronischer koronarer Herzkrankheit – keinen Bezug des Stimulationsergebnisses zur prognostischen Aussage, jedoch einen Bezug zur linksventrikulären Funktion beschrieb. M. Borggrefe und G. Breithardt [21] beschrieben in einer Studie, dass das Stimulationsergebnis mit hoher Spezifität Risikopatienten charakterisieren könne [21]. In diese Studie wurden sowohl Patienten in der frühen Postinfarktphase als auch in der chronischen Phase der KHK ohne dokumentierte ventrikuläre Tachykardien eingeschlossen. N. Treese und T. Meinertz [22] untersuchten 267 Patienten mit einer KHK mit und ohne Infarkt und fanden keine prognostische Aussage des Stimulationsergebnisses (241). In einer eigenen Studie an 100 Patienten mit KHK oder dilatativer Kardiomyopathie (DCM) konnte weder für die Patienten mit einer KHK noch mit einer DCM eine prognostische Aussage bestätigt werden [23]. Als Nebenbefund ergab diese Studie, dass die Induzierbarkeitsraten bei der DCM deutlich geringer sind als bei der KHK.

Nach dem aktuellen Wissensstand muss davon ausgegangen werden, dass das Ergebnis der programmierten ventrikulären Stimulation bei der KHK und der dilatativen Kardiomyopathie keine prognostische Aussage erlaubt.

Die Identifikation von primär asymptomatischen Menschen mittels der programmierten ventrikulären Stimulation ist nicht möglich.

Aus heutiger Sicht waren die Bemühungen, durch die programmierte Ventrikelstimulation eine Gruppe von Menschen mit einem erhöhten Risiko des plötzlichen Herztodes zu identifizieren, sehr wertvoll. Sie haben gezeigt, dass die primäre Risikostratifikation mit Hilfe eines invasiven, von der Spontanvariabilität unabhängigen Verfahrens nicht geeignet ist, diese Aufgabe zu erfüllen.

Übrig bleibt, dass die reproduzierbare Induzierbarkeit einer anhaltenden monomorphen ventrikulären Tachykardie bei der KHK ein spezifisches Ergebnis darstellt.

Die aktuellen ESC/DGK-Leitlinien nehmen hierauf Bezug. Wenn es um die Abklärung einer arrhythmiebezogenen Symptomatik geht und eine elektrophysiologische Untersuchung mit einem derartigen Ergebnis erfolgt, wird heute eine invasive therapeutische Maßnahme erwogen. Sei es eine ICD-Implantation oder eine katheterablative Therapie.

Inwieweit hierdurch eine Prävention des plötzlichen Herztodes möglich ist, bleibt bei den wissenschaftlichen Bemühungen.

## Antiarrhythmische Therapiekontrolle durch die serielle elektrophysiologische Testung

Die serielle ventrikuläre Stimulation in der Therapiekontrolle ventrikulärer Arrhythmien war in den 80er- und 90er-Jahren des vergangenen Jahrhunderts ein die Kongresse bestimmendes wissenschaftliches Thema.

Antitachykarde Schrittmacher, implantierbare Kardioverter-Defibrillatoren befanden sich auf dem Weg, die Katheterablation bestimmter ventrikulärer Tachykardien – zumindest nicht unter ICD-Schutz – galt als verwerflich.

Es blieb die pharmakologische antiarrhythmische Therapie. Viele neue Antiarrhythmika kamen und beherrschten den klinischen und wissenschaftlichen Markt: d/l Sotalol, d‑Sotalol, Propafenon, Flecainid, Mexiletin, Disopyramid und – wenn auch mit Einschränkungen aufgrund der damals noch zu klärenden Kinetik – Amiodaron. Das pharmakologische Interesse richtete sich insbesondere auf neue Klasse-III-Antiarrhythmika mit kürzerer Halbwertszeit und natürlich einem geringeren Nebenwirkungsprofil als Amiodaron.

Die *antiarrhythmische Effizienz* dieser Antiarrhythmika wurde in seriellen Testungen bestätigt oder in Frage gestellt. Die *Leerstimulation* ergab die Induzierbarkeit und den Induktionsmodus. Danach wurde das Pharmakon verabreicht und nach entsprechender Aufsättigung und Erhaltungsdosis bei therapeutischem Plasmaspiegel die Stimulation wiederholt. Bei fehlender Induktion galt das Pharmakon als effizient. Eine *erschwerte* Induktion (d. h. bei mindestens mehr als zwei weiteren aggressiveren Stimulationsmodi) wurde ebenfalls eine antiarrhythmische Effektivität gesehen. Da die Stimulationen eine erneute Platzierung des Stimulationskatheters notwendig machten, wurde für die in der Regel 3 bis 4 Tage geltende Aufsättigungsphase der Stimulationskatheter nach einem pharmakologisch definierten zeitlichen Intervall neu platziert oder -als „indwelling“ Katheter für diesen Zeitraum im Patienten belassen.

Zu den Klasse-I-Antiarrhythmika liegen eine Reihe von Daten mit unterschiedlichen, in der Regel kurzen Nachbeobachtungszeiten vor. Eine Vielzahl von Daten wurde zu diesem Thema publiziert. Kim et al. 1986 [24], Roy et al. 1983 [25] und Skale et al. 1986 [26] konnten zeigen, dass die Rezidiv- und plötzliche Herztodesrate zwischen 32 und 100 % innerhalb von 8 bis 22 Monaten Nachverfolgungszeit lag.

Langfristig konnte keine signifikante Senkung der Raten für Rezidive/ plötzlichen Herztod erfolgen. Die Daten zu Amiodaron als einzig verfügbarem Klasse-III-Antiarrhythmikum ergaben bei relativ hoher Induzierbarkeit der Arrhythmie – in der Regel einer anhaltenden monomorphen VT – innerhalb von Nachbeobachtungszeiten von 12 bis 25 Monaten Rezidivraten von 40 %. Die Diskussion darüber, inwieweit Amiodaron durch seine besondere Pharmakokinetik überhaupt im Rahmen einer seriellen Testung beurteilbar war, zeigte keine Ergebnisse [27].

Aus heutiger Sicht waren die Effizienzdaten unter Amiodaron besser als bei allen anderen Pharmaka.

Da im Rahmen der Weiterentwicklung der antiarrhythmischen Therapieformen immer mehr nichtpharmakologische Verfahren attraktiver wurden, verschwand die serielle Testung und damit auch die Pharmakotherapie aus der klinischen Routine.

## Hat die programmierte ventrikuläre Stimulation eine Zukunft?

Wo hat die programmierte Ventrikelstimulation heute ihre Bedeutung? Sie besteht in der Ursachenabklärung möglicher arrhythmiebedingter Symptome, unter Berücksichtigung der kardialen Grundkrankheit und der Art des Stimulationsergebnisses, z. B. KHK, monomorphe anhaltende VT; fraglich bei anderen Stimulationsergebnissen wie reproduzierbarem Kammerflimmern, polymorphen VT, allen Formen nichtanhaltender VT.

Möglicherweise kann dieses Verfahren in der Primärprävention des plötzlichen Herztodes bei der KHK jedoch eine Renaissance erleben: Sollte bei Patienten mit eingeschränkter LV-Funktion und KHK eine ICD-Implantation erwogen werden und eine anhaltende monomorphe VT induziert werden, könnte das in die Entscheidung für eine ICD-Implantation mit eingehen. Möglicherweise wäre eine derartige Entscheidung bei einer reproduzierbar induzierbaren polymorphen VT oder Kammerflimmern auch denkbar.

Hierzu müssen noch wissenschaftliche Daten erhoben werden. Interessant wäre ein solches Vorgehen bei einer Patientengruppe, deren Risiko für plötzlichen Herztod bisher nicht untersucht wurde: Die Patienten mit gering/leicht reduzierter LVEF von 40–60 %.

## Persönlicher Nachtrag

Als die programmierte ventrikuläre Stimulation (PVS) Anfang der 1980er-Jahre als neues Verfahren in die kardiologische Klinik eingeführt werden sollte, beurteilte die interventionell tätige kardiologische Oberärztin, der ich zugeteilt war: Was soll das? Wir sind doch froh, dass die Patienten solche Arrhythmien nicht spontan haben und jetzt sollen wir sie auch noch auslösen? Ein anderer bekannter interventionell sehr erfolgreicher Kardiologe beurteilte meine ersten Katheterablationen als eine „Tätigkeit, bei der sehr viel Dampf aufsteigt“. Diese Äußerung ergab sich aus den ersten Katheterablationen, die sowohl mit Radiofrequenzwechselstrom (RF; HAT 100, 200 Fa. Osypka) als auch mit Gleichstrom durchgeführt wurden. Primär wurde in unserem Göttinger Zentrum der Einsatz von RF präferiert, auch bei den großen VT-Kollektiven [28, 29].

Beide genannten Kardiologen haben später die interventionellen elektrophysiologischen Aktivitäten gefördert.
